# The implication of a translational triage tool in mass casualty incidents: part three: a multinational study, using validated patient cards

**DOI:** 10.1186/s13049-023-01128-3

**Published:** 2023-11-28

**Authors:** Amir Khorram-Manesh, Eric Carlström, Frederick M. Burkle, Krzysztof Goniewicz, Lesley Gray, Amila Ratnayake, Roberto Faccincani, Dinesh Bagaria, Phatthranit Phattharapornjaroen, Mohammed A. S. Sultan, Carl Montán, Johan Nordling, Shailly Gupta, Carl Magnusson

**Affiliations:** 1 Department of Surgery, Institute of Clinical Sciences, Sahlgrenska Academy, Gothenburg University, 405 30 Gothenburg, Sweden; 2 Gothenburg Emergency Medicine Research Group (GEMREG), Sahlgrenska Academy, 413 45 Gothenburg, Sweden; 3 Institute of Health and Care Sciences, Sahlgrenska Academy, Gothenburg University, 405 30 Gothenburg, Sweden; 4 Center for Disaster Medicine, Gothenburg University, 405 30 Gothenburg, Sweden; 5 USN School of Business, University of South-Eastern Norway, 3199 Borre, Norway; 6 Woodrow Wilson International Center for Scholars, Washington, DC USA; 7 Department of Security, Polish Air Force University, Dęblin, Poland; 8 Department of Primary Health Care & General Practice, University of Otago, Wellington, New Zealand; 9 Joint Centre for Disaster Research, Massey University, Wellington, New Zealand; 10 Sri Lanka Army Hospital, Narahenpita, Colombo, 08 Sri Lanka; 11 Emergency Department, Humanitas Mater Domini, 210 53 Castellanza, Italy; 12 Division of Trauma Surgery & Critical Care, J.P.N. Apex Trauma Center, All India Institute of Medical Sciences, New Delhi, India; 13 Department of Emergency Medicine, Faculty of Medicine Ramathibodi Hospital, Mahidol University, 10400 Bangkok, Thailand; 14 Emergency Department, King Khalid Hospital, Narjan, Saudi Arabia; 15 Karolinska MRMID—International Association for Medical Response to Major Incidents, Stockholm, Sweden; 16 Disasters, and Vascular Surgery, Department of Molecular Medicine and Surgery, Karolinska Institute, Stockholm, Sweden; 17 Department of Molecular and Clinical Medicine, Sahlgrenska Academy, Gothenburg University, 405 30 Gothenburg, Sweden

**Keywords:** Disaster, Feasibility, Mass casualty, Primary triage, Translational triage tool

## Abstract

**Background:**

Mass casualty incidents (MCI) pose significant challenges to existing resources, entailing multiagency collaboration. Triage is a critical component in the management of MCIs, but the lack of a universally accepted triage system can hinder collaboration and lead to preventable loss of life. This multinational study uses validated patient cards (cases) based on real MCIs to evaluate the feasibility and effectiveness of a novel Translational Triage Tool (TTT) in primary triage assessment of mass casualty victims.

**Methods:**

Using established triage systems versus TTT, 163 participants (1575 times) triaged five patient cases. The outcomes were statistically compared.

**Results:**

TTT demonstrated similar sensitivity to the Sieve primary triage method and higher sensitivity than the START primary triage system. However, the TTT algorithm had a lower specificity compared to Sieve and higher over-triage rates. Nevertheless, the TTT algorithm demonstrated several advantages due to its straightforward design, such as rapid assessment, without the need for additional instrumental interventions, enabling the engagement of non-medical personnel.

**Conclusions:**

The TTT algorithm is a promising and feasible primary triage tool for MCIs. The high number of over-triages potentially impacts resource allocation, but the absence of under-triages eliminates preventable deaths and enables the use of other personal resources. Further research involving larger participant samples, time efficiency assessments, and real-world scenarios is needed to fully assess the TTT algorithm's practicality and effectiveness in diverse multiagency and multinational contexts.

## Introduction

Mass casualty incidents (MCI) differ from multi-casualty incidents by distinguishing the number of casualties versus available resources. MCI is characterized by limited resources for affected victims, necessitating the use of MCI triage to put order into the chaos by differentiating between noncritically and critically injured casualties. Since major incidents and MCI are heterogeneous by nature, they cause diverse impacts, injury panorama, and outcomes. Consequently, their unexpectedness favors an "all-hazards" approach [1]. Triage begins at the scene of an incident, i.e., prehospital level. Primary triage occurs in the field and aims to determine priorities for treatment on scene and transport to the hospital. Primary triage will be completed with a secondary triage at the casualty clearing station at the site of a major incident and often before transport to a hospital [1, 2].

There are several prehospital triage systems but no consensus on what constitutes the optimal choice. This heterogeneity constitutes a threat in a mass casualty incident (MCI) in which triage is used during multiagency collaboration to prioritize casualties according to the injuries’ severity [2–5]. A major difference between ordinary triage of patients at hospitals´ emergency departments (ED) and utilitarian (the best for the most) prehospital triage during disasters and public health emergencies (DPHEs) lies in the available resources in the respective areas, which also may explain the variations in triage systems globally [6–12]. However, in both triage systems four factors, i.e., speed, precision, fairness, and compatibility are of critical value [3–5, 12]. While precision might be prioritized to define a diagnosis in a resource-rich environment, such as in an ED with more time and interventional resources (e.g., laboratory, blood pressure measurements), speed is crucial in a prehospital setting, particularly in MCI, when resource availability and time are limited [3, 5–8]. Consequently, the hastier the triage, the bigger the risk for faulty categorization, represented as under or over-triage [3–8].

Under-triage increases the mortality among casualties because of a prolonged time to establish an accurate diagnosis and proper interventions. Over-triage, on the other hand, results in the unnecessary use of resources, and the likelihood of increased morbidity [3, 5, 13, 14]. As such, prehospital triage systems allow for a more limited precision, especially at the primary line of triage since speed remains a priority while hospital triage can often sacrifice time to maximize precision. Fairness in prehospital triage is achieved by assessing patients objectively according to a set of parameters of vital signs or mechanisms of injury, and not discriminating in terms of age, gender, nationality, religion, or any other individual aspect [3, 5, 6]. Compatibility applies to triage systems being translational across agencies and to pre-hospital systems being able to integrate seamlessly with their hospital counterparts in terms of categorization, etc. [3, 7, 11–14].

**Fig. 1:**
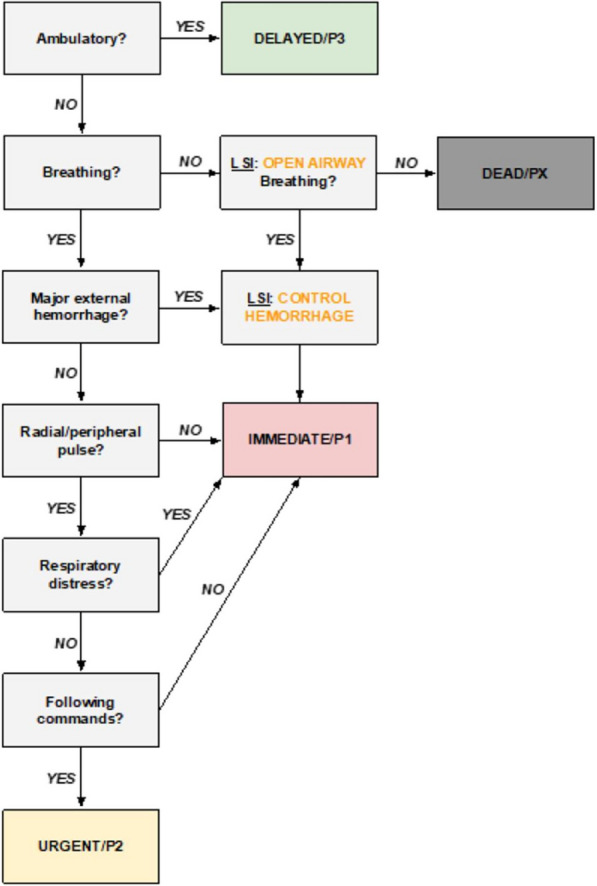
System constructed from majority criteria, modified according to discussion regarding criteria and lifesaving interventions (LSIs) [4]

Earlier studies have confirmed the feasibility of using a Translational Triage Tool (TTT), resulting from the sum of several primary prehospital triage systems, which could enable the translation of these triage systems into one (Fig. 1) [3]. Undertaking a Delphi study, a group consisting of several trauma and emergency medicine experts, reviewed and validated the tool, confirming the feasibility of this tool/algorithm with minimal necessary revisions. The tool primarily aims to clarify the need for interventions and guide healthcare providers to implement what is medically necessary for decisive and critical steps in managing a major incident with multiple casualties of different severity [5]. Additionally, Khorram-Manesh et al. [5] achieved a consensus among medical experts about the ratio of three (n = 3) casualties or more per healthcare provider to define the need for a primary triage algorithm. According to participating medical experts, they could preferably manage only two victims at a time.

After presenting the tool [3], and expert evaluation [5], a pilot study to verify its clinical efficiency was considered as the next step. However, since disasters and MCIs are rare, and ordinary incidents are not serious and time-constrained enough to stress the severity of a situation and utilization of the tool, testing the tool on validated patient cards, representing diverse medically affected victims was suggested as an attractive alternative solution [15–18]. The current study aimed to evaluate the feasibility and outcomes of the TTT algorithm in an MCI, compared to established national and institutional triage systems, by using several fictive patients (i.e., validated cards) among a multinational group of participants. The quantitative data analysis will be supplemented by qualitative feedback from participants to the country leaders after each test in verbal or written debrief comments.

## Material and methods

### Patient cases

For this study, authentic patient cases representing victims of real MCIs have been collected and made into patient cards, utilized in disaster management courses [15–18]. The Medical Response to Major Incident (MRMI) is a module-based simulation course that uses authentic and scientifically validated patient cards (MACSIM®), derived from victims of MCIs [19]. The patients have a necessary medical history, critical input, and output data for prehospital and hospital assessments [15–18].

**Fig. 2:**
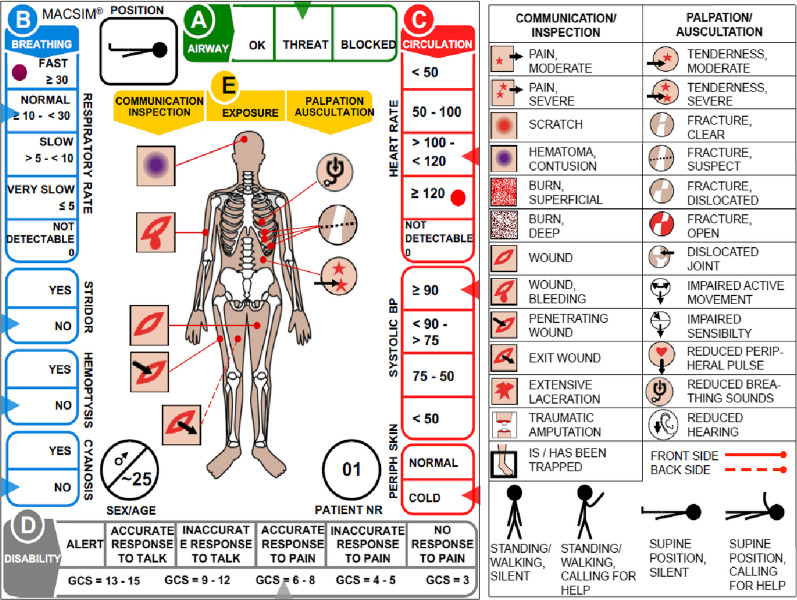
Shows a victim with multiple injuries and a list of diverse information to explain the signs and define the injuries on the right side [15–19]

Five patient cards were selected by the representative for the MACSIM® system The study administrators had no involvement in this selection. Five patient cards were chosen because of the results of the previous study, in which the number of patients per healthcare provider defining an MCI was set to three [5]. It was assumed that five patients were enough to create an MCI, although no time pressure could be applied to the participants due to the study design. Figure 2 shows all MACSIM® patients, which were given to each participant. Participants received a normal MACSIM card initially to familiarize themselves with the characteristics of the card, and relevant signs before triaging the study´s five patients (Appendix A, Appendix B, Table 1).

**Table 1:** Patient numbers, characteristics, and triage color codes

Pat. no	Characteristics	Triage code
1	Thirty years old man, supine position, airway ok, breathing > 30, reduced breathing sound left side, no stridor, hemoptysis, no cyanosis, HR > 100-< 120, SBP ≥ 90, skin temp normal, GCS 9–12. Injured right ear, posterior and anterior burn injury both legs, and multiple wounds	RED
2	Fifty-two years old man, supine position, airway ok, breathing slowly > 5- < 10, no stridor, no hemoptysis, no cyanosis, HR 50–100, SBP ≥ 90, skin temp normal, GCS 3. Multiple bone and rib injuries, skull hematoma contusion, and right eye injury	RED
3	Twenty-six years old female, supine position, asks for help, airway ok, breathing > 30, reduced breathing sound left side, no stridor, no hemoptysis, no cyanosis, HR ≥ 120, SBP ≥ 90, Skin cold, GCS 13–15, multiple bone injuries and ribs fractures	YELLOW
4	Forty-two years female, supine position, asks for help, airway ok, breathing normal, no stridor, no hemoptysis, no cyanosis, HR > 100—< 120, SBP ≥ 90, Skin cold, GCS 9–12, eye contusion on the right side, multiple bone injuries and no palpable pulse on left radialis	YELLOW
5	Forty-three years female, supine position, asks for help, airway ok, breathing normal, no stridor, no hemoptysis, no cyanosis, HR > 100—< 120, SBP ≥ 90, Skin normal, GCS 13–15, multiple wounds on the neck, both arms and legs	GREEN

### Participants countries

The researchers actively engaged in a multinational research group, Research Alliance in Disaster and Emergency Medicine (RADEM) were contacted regarding possible participation. Additionally, researchers from countries previously engaged in the evaluation of TTT were invited. Purposefully, these commonly accepted subject-matter experts recruited new participants from their research networks. Although the current study aimed to compare the results of the traditionally used primary triage model in participating centers with TTT, the type of triage system used in each country was not an inclusion criterion.

### Inclusion criteria

Physicians, nurses, and paramedics actively engaged in primary triage in the fields of prehospital, emergency care, and or disaster medicine. Staff with no experience in triage procedures were excluded.

### The number of participants and participating countries

Representatives from eight countries (India, Italy, New Zealand, Poland, Saudi Arabia, Sri Lanka, Sweden, and Thailand), called subject-matter specialists, joined the study, and together recruited 164 participants from their countries. One of the participants did not conduct the second round, thus, was excluded. Another 5 participants were excluded due to incomplete data (Table 2).

**Table 2:** Showing the demography of participants regarding the profession, years of experience, how they familiarized themselves with TTT, and the time between the two tests

Country	No. of participants	Participants Background	Years of experience	Learning about TTT	The time between the 1st and the 2nd tests
India	23	17 physicians (15 trauma or emergency physicians and 2 general surgeons), and 6 Nurses	5–15	Written and verbal information and email follow-up	3–4 weeks
Italy	40	12 Physicians, 20 Nurses, 4 Administrators, and 4 Surgical residents	1–20	Written and verbal information and email follow-up	2 days
New Zealand	13	3 physicians, 2 nurses, and 8 paramedics	More than 5	Written information and email follow-up	1–2 weeks
Poland	16	16 Physicians	More than 10	Written and verbal information and email follow-up	10 days
Saudi Arabia	15	5 physicians, 7 Nurses, and 3 paramedics	More than 5	Written and verbal information and email follow-up	2–3 weeks
Sri Lanka	10	5 military physicians, and 5 military nurses	More than 10	Written and verbal information and email follow-up	1–2 weeks
Thailand	10	6 Emergency Physicians, 1 Emergency Nurse, and 2 Paramedics		Written and verbal information and email follow-up	2 weeks
Sweden	31	2 Physicians (Surgery 1, Anesthesiology 1), 2 Residents (Emergency Med and Anesthesiology), 27 Nurses (17 prehospital, 3 ICU/anesthesiology, 7 ordinary nurses)	4–35	Written and verbal information and email follow-up	2–3 weeks

### Study process

Each subject-matter specialist recruiting national participants for the study was informed regarding the study and received information about MACSIM® cards, and how to interpret the cards [15–19]. Moreover, they were informed about the study design and the period of study (November 2022-March 2023). Participants were not familiar with TTT and received lectures, face-to-face information, and/or written information. They had opportunities to test the tool and ask questions from each country leader. Each participant had to triage five (5) patients (numbered 1 to 5) by their national or institutional primary triage system in the first round and the TTT tool in the second round (the same patient cards). The time between the two assessments varied between 2 and 21 days (Table 2). This deliberate spacing aimed to serve the purpose of a washout period, ensuring that the effects of prior triage experiences did not influence subsequent assessments. Furthermore, neither the participants nor the country leaders were informed about the triage outcomes/keys. The results of each round were compiled and transferred to an Excel file and sent to the lead author. Each participant received full information regarding the study and how to participate and act in each round of the study. The results obtained after two triage rounds were to be statistically analyzed by one of the authors, with experience in statistical analyses, using validated statistical methods.

Each country leader was asked to comment on the performance of the participants and the feasibility and ease of understanding and performing the triage, using TTT. Participants could comment on the tool and its feasibility, and any concerns experienced during the trial. These qualitative data were collected by each country leader and were sent to the lead author for analysis and further assessment.

### Statistical analysis

The statistical analysis was pursued to observe similarities or differences between the two systems (i.e., own system vs. TTT). Thus, testing whether one mutual triage system (TTT) can be used as a universal system. Professional statisticians validated the process and results. The diagnostic accuracy of the triage systems in the first round and TTT in the second round were assessed using a 2 × 2 contingency table. Cases were dichotomized into two groups: Immediate/Red/P 1 and non-immediate. The sensitivity, specificity, accuracy, positive predictive value (PPV), negative predictive value (NPV), over-triage, under-triage, positive likelihood ratio (LR+), and negative likelihood ratio (LR-) were calculated as compared with the reference standard, i.e., the outcome and the corresponding pre-determined color of the MACSIM® cards unknown to the respondents. The over-triage was calculated as 1- PPV based on the Cribari matrix method whereas under-triage rates were calculated as 1-sensitivity [20]. Wilson's method was used to calculate the 95% confidence intervals (CI) for all estimates of diagnostic accuracy (Tables 3, 4).

**Table 3:** Performance of Triage Systems in round one and TTT in round two

System	Sensitivity	Specificity	Accuracy	PPV	NPV	Over-triage (1-PPV)^2^	Under-triage (1-Sens)^3^	LR+	LR−
Sieve	0,983	0,736	0,834	0,713	0,985	0,288	0,017	3,717	0,023
	[0.939,0.995]^1^	[0.666,0.796]	[0.787,0.873]	[0.638,0.777]	[0.946,0.996]	[0.223,0.362]	[0.005,0.061]	[2.898,4.769]	[0.006,0.093]
TTT	1	0,368	0,621	0,513	1	0,487	0	1,582	0
	[0.968,1]	[0.3,0.442]	[0.564,0.675]	[0.448,0.578]	[0.943,1]	[0.422,0.552]	[0,0.032]	[1.412,1.772]	[0,0.188]
START	0,917	0,722	0,8	0,688	0,929	0,312	0,083	3,3	0,115
	[0.857,0.953]	[0.656,0.78]	[0.754,0.84]	[0.616,0.751]	[0.877,0.96]	[0.249,0.384]	[0.047,0.143]	[2.621,4.155]	[0.065,0.205]
TTT	0,992	0,657	0,791	0,658	0,992	0,342	0,008	2,89	0,012
	[0.958,0.999]	[0.588,0.719]	[0.744,0.831]	[0.59,0.721]	[0.958,0.999]	[0.279,0.41]	[0.001,0.042]	[2.382,3.505]	[0.002,0.082]
Mixed	0,773	0,495	0,606	0,505	0,766	0,495	0,227	1,53	0,459
	[0.658,0.857]	[0.399,0.592]	[0.53,0.677]	[0.409,0.6]	[0.649,0.853]	[0.4,0.591]	[0.143,0.342]	[1.21,1.935]	[0.282,0.748]
TTT	0,955	0,444	0,648	0,534	0,936	0,466	0,045	1,718	0,102
	[0.875,0.984]	[0.35,0.543]	[0.573,0.717]	[0.444,0.621]	[0.828,0.978]	[0.379,0.556]	[0.016,0.125]	[1.43,2.065]	[0.033,0.316]

*PPV* positive predictive value, *NPV* negative predictive value, *LR+* positive likelihood ratio, *LR−* negative likelihood ratio

^1^95%—Confidence intervals

^2^Over-triage calculated as 1- Positive predictive value according to the Cribari matrix

^3^Under-triage calculated as 1- Sensitivity according to the Cribari matrix

**Table 4:** Diagnostic accuracy per country and system in the first round compared to TTT in the second round

Country	System	Sensitivity	Specificity	Accuracy	PPV	NPV	Over-triage (1-PPV)^2^	Under-triage (1-Sens)^3^	LR+	LR−
**A**	START	0.975	0.775	0.855	0.743	0.979	0.257	0.025	4.333	0.032
		[0.913,0.993]^1^	[0.692,0.841]	[0.8, 0.897]	[0.652,0.817]	[0.926,0.994]	[0.183,0.348]	[0.007,0.087]	[3.103,6.051]	[0.008,0.127]
**A**	TTT	1	0.667	0.8	0.667	1	0.333	0	3	0
		[0.954,1]	[0.578,0.745]	[0.739,0.85]	[0.578,0.745]	[0.954,1]	[0.255,0.422]	[0,0.046]	[2.329,3.864]	[0,0.149]
**B**	START	0.812	0.729	0.762	0.667	0.854	0.333	0.188	3	0.257
		[0.647,0.911]	[0.59,0.834]	[0.659,0.842]	[0.51,0.794]	[0.716,0.931]	[0.206,0.49]	[0.089,0.353]	[1.832,4.912]	[0.122,0.54]
**B**	TTT	1	0.625	0.775	0.64	1	0.36	0	2.667	0
		[0.893,1]	[0.484,0.748]	[0.672,0.853]	[0.501,0.759]	[0.886,1]	[0.241,0.499]	[0,0.107]	[1.851,3.842]	[0,0.395]
**C**	SIEVE	1	0.911	0.947	0.882	1	0.118	0	11.25	0
		[0.886,1]	[0.793,0.965]	[0.871,0.979]	[0.734,0.953]	[0.914,1]	[0.047,0.266]	[0,0.114]	[4.415,28.668]	[0,0.286]
**C**	TTT	1	0.578	0.747	0.612	1	0.388	0	2.368	0
		[0.886,1]	[0.433,0.71]	[0.638,0.831]	[0.472,0.736]	[0.871,1]	[0.264,0.528]	[0,0.114]	[1.683,3.333]	[0,0.456]
**D**	SIEVE	0.984	0.656	0.787	0.656	0.984	0.344	0.016	2.859	0.025
		[0.914,0.997]	[0.555,0.745]	[0.716,0.844]	[0.555,0.745]	[0.914,0.997]	[0.255,0.445]	[0.003,0.086]	[2.156,3.792]	[0.003,0.173]
**D**	TTT	1	0.258	0.555	0.473	1	0.527	0	1.348	0
		[0.942,1]	[0.18,0.355]	[0.476,0.631]	[0.39,0.558]	[0.862,1]	[0.442,0.61]	[0,0.058]	[1.196,1.519]	[0,0.505]
**E**	MIXED	0.739	0.333	0.496	0.425	0.657	0.575	0.261	1.109	0.783
		[0.597,0.844]	[0.234,0.451]	[0.406,0.586]	[0.323,0.534]	[0.492,0.792]	[0.466,0.677]	[0.156,0.403]	[0.873,1.409]	[0.434,1.412]
**E**	TTT	0.909	0.47	0.645	0.533	0.886	0.467	0.091	1.714	0.194
		[0.788,0.964]	[0.354,0.588]	[0.553,0.729]	[0.422,0.642]	[0.74,0.955]	[0.358,0.578]	[0.036,0.212]	[1.341,2.191]	[0.073,0.51]
**F**	MIXED	0.769	0.692	0.723	0.625	0.818	0.375	0.231	2.5	0.333
		[0.579,0.89]	[0.536,0.814]	[0.604,0.817]	[0.453,0.771]	[0.656,0.914]	[0.229,0.547]	[0.11,0.421]	[1.493,4.187]	[0.16,0.693]
**F**	TTT	1	0.487	0.692	0.565	1	0.435	0	1.95	0
		[0.871,1]	[0.339,0.638]	[0.572,0.791]	[0.422,0.698]	[0.832,1]	[0.302,0.578]	[0,0.129]	[1.436,2.648]	[0,0.626]
**G**	SIEVE	1	0.733	0.84	0.714	1	0.286	0	3.75	0
		[0.839,1]	[0.556,0.858]	[0.715,0.917]	[0.529,0.847]	[0.851,1]	[0.153,0.471]	[0,0.161]	[2.072,6.788]	[0,0.531]
**G**	TTT	1	0.4	0.64	0.526	1	0.474	0	1.667	0
		[0.839,1]	[0.246,0.577]	[0.501,0.759]	[0.373,0.675]	[0.758,1]	[0.325,0.627]	[0,0.161]	[1.244,2.232]	[0,0.999]
**H**	MIXED	0.9	0.6	0.72	0.6	0.9	0.4	0.1	2.25	0.167
		[0.699,0.972]	[0.423,0.754]	[0.583,0.825]	[0.423,0.754]	[0.699,0.972]	[0.246,0.577]	[0.028,0.301]	[1.418,3.571]	[0.043,0.641]
**H**	TTT	1	0.533	0.72	0.588	1	0.412	0	2.143	0
		[0.839,1]	[0.361,0.698]	[0.583,0.825]	[0.422,0.736]	[0.806,1]	[0.264,0.578]	[0,0.161]	[1.462,3.141]	[0,0.739]

*PPV* positive predictive value, *NPV* negative predictive value, *LR+* positive likelihood ratio, *LR−* negative likelihood ratio

^1^95%—Confidence intervals

^2^Over-triage calculated as 1-Positive predictive value according to the Cribari matrix

^3^Under-triage calculated as 1- Sensitivity according to the Cribari matrix

Additionally, a statistical analysis to assess the diagnostic systems in terms of over-triage, under-triage, and accuracy was conducted, using a linear mixed effect model (lmer4). This assured accountability for the correlation between observations within these groups, including countries and patient cases as random effects to account for any variability between triage systems in terms of their diagnostic performance. Two models were fitted, one comparing TTT in the second round with all national/institutional systems in the first round. Secondly, a per-system comparison between TTT and the most occurrent triage systems. *P*-values < 0.05 were considered statistically significant for fixed effects i.e., prediction of outcome for TTT. All statistical analyses were performed using R software version 4.2.2 (R Foundation for Statistical Computing, Vienna, Austria).

### Ethics

The study follows Swedish law's ethical guidelines and principles and is exempted from ethical approval requirements. In Sweden, where the study is conducted, ethical approval is mandatory if the research includes sensitive data on the participants such as race, ethnical heritage, political views, religion, sexual habits, and health or physical interventions or employs a method that aims to affect the person physically or psychologically [21]. Written and verbal information, including the study’s design, purpose, and data use, were provided for each participant. However, some countries required their ethical approval: The University of Otago, Wellington, ref D22/350 issued New Zealand ethics approval. Thailand ethical approval was issued by Mahidol University, Bangkok, ref MURA 2022/753.

## Results

### General results

There were several institutional, and national triage systems. The most used systems were Sieve and START triage, followed by other systems (the latter were grouped as one, called Mixed). Sieve and START are both recognized processes for prioritizing victims of MCIs.

### Statistical analysis

Five patient cards were triaged by 164 participants (one missed the second round) on two rounds, resulting in 1575 triaged cases, of which, 790 cases were in the first round and n = 785 in the second round with TTT (n = 5 missing). START triage was used in n = 330 cases, Sieve in n = 290 cases, and in 170 cases mixed triage systems were used. The TTT algorithm had similar sensitivity as the Sieve primary triage, 100% (95% CI 96.8–100), and 98% (95% CI 93.9–99.5), respectively but a higher sensitivity than the START primary triage, 99.2% (95% CI 95.8–99.9) vs. 91.7% (95% CI 85.7–95.3). The TTT algorithm had a lower specificity, compared to Sieve, 36.8% (95% CI 30.0–44.2) vs. 73.6% (95% CI 66.6–79.6), whereas START triage systems used in the first round had a specificity of 77.2% (95% CI 65.6–78.0) compared to 65.7% (95% CI 58.8–71.9) for TTT. The positive predictive value (PPV) was lower for TTT at 51.3% (95% CI 44.8–57.8) compared with Sieve’s 71.3% (95% CI 63.8–77.7). PPV for the START triage system was 68.8% (95% CI 44.8–57.8), compared to 65.8% (95% CI 59.0–72.1) for TTT. Consequently, a higher rate of false positives for the TTT algorithm yielded an over-triage for the TTT of 48.7% (95% CI 42.2–55.2), compared to Sieve at 28.8% (95% CI 22.3–36.2). For countries using START in the first round TTT yielded an over-triage of 34.2% (95% CI 27.9–41.0) vs. 31.2% (95% CI 24.9–38.4) for START. Regarding under-triage, the TTT had almost zero under-triage (0–0.8%) in the largest Sieve and START group (Table 3). In the mixed system group, TTT had an under-triage of 4.5% but out of the three countries with mixed systems, the finding originated from one country, which had a relatively high misclassification rate overall of 10–26.1% under-triage (Table 4).

A variation in diagnostic accuracy was found between countries for the TTT algorithm. However, out of eight countries the TTT yielded a 100% sensitivity in all but one country, where TTT showed a sensitivity of 90.9%. The range of specificity was between 25.5 and 66.7% (Table 4). Most of the triage systems, and the TTT algorithm had good accuracy for triaging patients 1, 2, and 4. However, patients 3, and 5 caused some issues, leading to over-triage in almost all systems. When adjusting for the variance of country and patients regarding over-triage, under-triage, and accuracy we found that TTT in round two had a significantly higher over-triage compared to all other systems in the first round (estimate = 1.565, SE = 0.198, z = 7.904, *p* < 0.001). Under-triage was significantly lower for TTT compared to all triage systems (estimate = −1.828, SE = 0.407, z = −4.493, *p* < 0.001). Moreover, the accuracy for TTT was found to be significantly lower (estimate = -0.812, SE = 0.161, z = −5.055, *p* < 0.001). In- between system comparisons, the TTT algorithm had a higher over-triage as compared to Sieve (estimate = 3.2758, SE = 0.4391, z = 7.461, *p* < 0.001). There was no significant difference regarding under-triage (estimate = -17.523, SE = 1716, z = −0.01, *p* = 0.992). TTT had a significantly lower accuracy than Sieve triage (estimate = −2.774, SE = 0.388, z = −7.152, *p* < 0.001). For those countries (n = 5) using START in the first round, the TTT algorithm had a significantly higher over-triage (estimate = 1.036, SE = 0.350, z = 2.964, *p* < 0.01) and a significantly lower under-triage (estimate = −2.250, SE = 0.768, z = −2.298, *p* < 0.01). There was no significant difference in accuracy between TTT and START (estimate = −0.189, SE = 0.270, z = −0.699, *p* = 0.484). TTT appears to be superior in terms of identifying critically ill patient cases with lower under-triage compared to triage systems used in this study but at the expense of a higher over-triage rate and thus a lower accuracy.

The statistical values in the tables, such as sensitivity, specificity, accuracy, PPV, negative predictive value, over-triage, and under-triage, are all presented with their corresponding 95% confidence intervals. Additionally, the positive and negative likelihood ratios (LR + and LR-) are included for each system and country, providing a comprehensive overview of the performance of each triage system in the study.

### Qualitative data

Almost all participants expressed that the TTT algorithm was easy to understand and follow, and it took a shorter time to assess each patient and perform the triage. According to each country leader´s report participants could easily use and understand the algorithm. Some participants expressed difficulties in using the blue tag for some of the patients. The Blue color corner tag in SMART triage is referred to as expectance or the fourth priority. These are immediate and severe cases that cannot be handled immediately due to the lack of resources and or the fact that they cannot survive despite the best available resources.

## Discussion

The outcomes of this study showed that Sieve triage appears to be superior to all other primary triage systems. The TTT algorithm matches Sieve’s primary triage in sensitivity and START primary triage in specificity. TTT is more sensitive than the START triage system but has lower specificity. These findings suggest that TTT captures the general characteristics of both commonly used primary triage systems and can be used as an alternative for primary triage. However, although promising, the use of the TTT algorithm results in a concerning over-triage, which may affect resource allocation. On the other hand, TTT does not need medical and instrumental interventions, such as blood pressure or heart/respiratory rate assessments, which could be an advantage in certain situations where such assessments are not possible or available, thus, offering a quicker triage performance, which can also be done by other staff than medical staff at the primary triage line [2–5, 22–25].

In a prehospital setting and as a primary triage tool, one should use an instrument that can work quickly with high sensitivity. Such an instrument allows over-triage since the main goal is to transfer the patient to the secondary triage areas or the closest hospital as soon as possible and without time-consuming diagnostic intervention [3, 5, 6, 26]. Consequently, the over-triage presented in this study as well as in other studies concerns only resource consumption. On the other hand, by using an algorithm, such as the TTT, with no under-triage, preventable deaths can be avoided, and the use of other staff categories can be facilitated. In extreme situations when every available human resource is crucial for the outcome, using other staff with lower competencies, non-governmental agencies with diverse backgrounds, and even laypersons can save lives [22, 25]. In such a situation, when flexibility in the management system is required [23, 24], the use of a simple, and easy-to-perform algorithm such as TTT cannot be underestimated.

In this study, no triage system seems to be better than the other, emphasizing the lack of consensus in using a specific triage system, especially in MCIs and when vulnerable patients are concerned [7, 27, 28]. Ultimately, the choice of triage system will depend on the specific context and the availability of the resources. In this perspective, an algorithm such as TTT might better substitute the traditional or established triage systems that independently claim to be best﻿ [26]. Using the initial concept of utilitarianism, the historical review emphasizes the rule of saving the most, and from a primary triage perspective, this process must proceed rapidly [24]. Therefore, there is no time or opportunity for discussions about a special color tag or confusion about the number of respiratory or heart rates counted, the measure obtained, or the age and gender of the victims [29]. This is especially important in an MCI, which presents a chaotic environment [29–31]. Consequently, a mutual, simple, and easy-to-perform triage algorithm that can initially close the gaps between different and existing prehospital primary triage systems is undoubtedly crucial. Prospective studies are needed to understand the strengths and weaknesses of the TTT compared to established triage systems in diverse environments and to verify the impacts of the algorithm in real-life events and more stressful situations.

One common "outlier to all triage methodologies” are children and the unappreciated narrowing pulse pressure despite an acceptable systolic blood pressure that will collapse suddenly leading to sudden death. This critical situation, which may affect all children in shock irrespective of etiology, is hard to recognize since the signs and symptoms of shock are not those of the underlying disease. Pulse and blood pressure cannot be used as diagnostic markers, and in several cases, the final diagnosis depends on the response to treatment and clinical suspicion. However, two common symptoms might be the deterioration of the respiratory system and confusion (mental affection), which both can be identified at the early stages of the TTT algorithm [32, 33].

This study evaluated the feasibility and outcomes of the TTT algorithm on several validated patient cards [15–18] in an MCI and among participants from several countries with different triage systems. The aim was not to present a new system in primary triage but rather a translational triage tool that can mutually be used to quickly avoid differences in priority settings, especially in a multiagency and multinational perspective. The participants’ general understanding, reported by most of the country leaders, was that participants could easily assess the patient cards and use and understand the algorithm. This was confirmed qualitatively by some participants, who commented that the TTT algorithm was easy to follow and took a shorter time to perform. One difficulty expressed by some participants in this study was the lack of a blue tag. Compared to the Sieve triage, neither START nor the new NHS Major Incident Triage Tool (MITT) has a blue tag [27, 29, 34]. Utilizing blue tags may simplify the decision-making between salvageable and non-salvageable RED patients. However, it also presents a new option, which can slow the triage process since medical interventions are needed to affirm the correct decision.

Finally, this study, again, emphasizes the need for a standardized primary triage system, since a variety of diverse triage systems in use is a real threat to the multiagency and multinational collaboration, which is a necessity in a globalized healthcare system with an increasing number of disasters and public health emergencies [35, 36]. Although other suggestions have been presented [24, 29, 36], so far TTT algorithm is the only research-based suggestion and might be one of the solutions.

### Limitations

Several limitations to this study should be acknowledged. First and foremost, the number of participants in the study was relatively small. In addition, the selection of the participants, people with knowledge of triage, may result in a selection bias of choosing those who may be more familiar/in favor of new tools such as TTT. A larger sample size and heterogeny population would provide a more robust statistical analysis and increase the generalizability of the findings. Second, the study did not measure the time taken to perform the triage procedures using the TTT algorithm compared to other triage systems. Time efficiency is an essential factor to consider when evaluating the effectiveness of a triage tool, particularly in mass casualty incidents where rapid decision-making is crucial. Third, the study utilized validated patient cards rather than real-life mass casualty incidents for triage evaluation. While this approach allows for a controlled comparison of the TTT algorithm and occurring triage systems, it may not fully capture the complexities and challenges that emerge in real-world scenarios. Fourth, the same cards were used on two different occasions, which might be considered as a bias. However, the deliberate spacing between the two tests was supposed to serve the purpose of a washout period, ensuring that the effects of prior triage experiences did not influence the next assessments. Furthermore, neither the participants nor country leaders knew the outcome of each card triage. Lastly, the study focused primarily on the feasibility of the TTT algorithm, with less emphasis on other factors such as resource allocation, patient flow, and inter-agency coordination. A comprehensive evaluation of the TTT algorithm should also consider the aforementioned factors to provide a more holistic understanding of its implications in MCIs. Future studies should explore these aspects in greater detail, preferably in a simulation exercise, to fully assess the potential benefits and drawbacks of implementing the TTT algorithm in disaster response efforts before its implementation in real-life events.

## Conclusions

In conclusion, the TTT algorithm appears to be a feasible and promising primary triage tool for MCIs. The high number of over-triages observed in this study may be acceptable given the absence of under-triage, which helps eliminate preventable deaths. Moreover, the increased resource utilization associated with over-triage could potentially be managed by involving other healthcare staff, non-governmental agencies personnel, and laypersons with lower triage competencies, due to the simple and easy-to-follow nature of the TTT algorithm. This flexibility in resource management can be particularly advantageous in extreme situations where every available human resource is crucial for optimizing outcomes. By addressing these limitations and building on the findings of this study, the TTT algorithm has the potential to serve as an effective, efficient, and universally applicable primary triage tool in mass casualty incidents, enhancing collaboration and decision-making across multiagency and multinational contexts.

## Figures and Tables

**Fig. 3:**
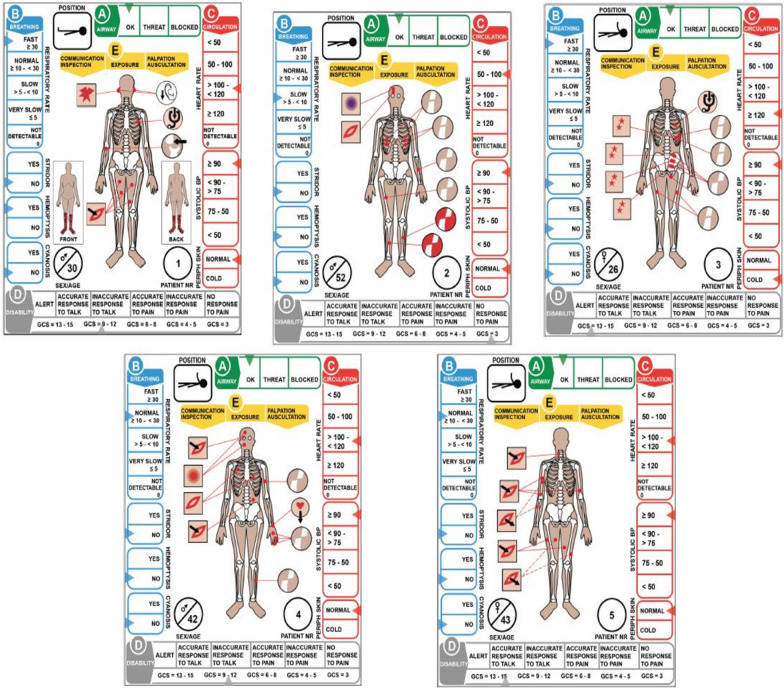
Shows all victims included in this study [15–19]

## Data Availability

All data generated or analyzed during this study are included in this published article. References used in the literature review are available on the Worldwide Web.

## Appendix A

Shows an example of a MACSIM® patient card [19] (Fig. 2).

## Appendix B

Shows all five victims included in this study [15–19] (Figs. 1, 2, 3).

## Abbreviations

ED: Emergency Departments
DPHE: Disasters and Public Health Emergencies
LSI: Life-Saving Interventions
LR +: Positive likelihood ratio
LR −: Negative likelihood ratio
MACSIM: Mass Casualty Simulation
MCI: Mass Casualty Incidents
MITT: Major Incident Triage Tool
NPV: Negative predictive value
PPV: Positive predictive value
RADEM: Research Alliance in Disaster and Emergency Medicine
START: Simple Triage and Rapid Transport
TTT: Translational Triage Tool
