# Correction: The implication of a translational triage tool in mass casualty incidents: part three: a multinational study, using validated patient cards

**DOI:** 10.1186/s13049-024-01185-2

**Published:** 2024-02-19

**Authors:** Amir Khorram-Manesh, Eric Carlström, Frederick M. Burkle, Krzysztof Goniewicz, Lesley Gray, Amila Ratnayake, Roberto Faccincani, Dinesh Bagaria, Phatthranit Phattharapornjaroen, Mohammed A. S. Sultan, Carl Montán, Johan Nordling, Shailly Gupta, Carl Magnusson

**Affiliations:** 1 Department of Surgery, Institute of Clinical Sciences, Sahlgrenska Academy, Gothenburg University, 405 30 Gothenburg, Sweden; 2 Gothenburg Emergency Medicine Research Group (GEMREG), Sahlgrenska Academy, 413 45 Gothenburg, Sweden; 3 Institute of Health and Care Sciences, Sahlgrenska Academy, Gothenburg University, 405 30, Gothenburg, Sweden; 4 Center for Disaster Medicine, Gothenburg University, Gothenburg, Sweden; 5 USN School of Business, University of South-Eastern Norway, 3199 Borre, Norway; 6 Woodrow Wilson International Center for Scholars, Washington, DC USA; 7 Department of Security, Polish Air Force University, Dęblin, Poland; 8 Department of Primary Health Care & General Practice, University of Otago, Wellington, New Zealand; 9 Joint Centre for Disaster Research, Massey University, Wellington, New Zealand; 10 Sri Lanka Army Hospital, 08 Narahenpita, Colombo, Sri Lanka; 11 Emergency Department, Humanitas Mater Domini, 210 53 Castellanza, Italy; 12 Division of Trauma Surgery & Critical Care, J.P.N. Apex Trauma Center, All India Institute of Medical Sciences, New Delhi, India; 13 Department of Emergency Medicine, Faculty of Medicine Ramathibodi Hospital, Mahidol University, 10400 Bangkok, Thailand; 14 Emergency Department, King Khalid Hospital, Narjan, Saudi Arabia; 15 Karolinska MRMID—International Association for Medical Response to Major Incidents, Karolinska Institute, Stockholm, Sweden; 16 Disasters, and Vascular Surgery, Department of Molecular Medicine and Surgery, Karolinska Institute, Stockholm, Sweden; 17 Department of Molecular and Clinical Medicine, Sahlgrenska Academy, Gothenburg University, 405 30 Gothenburg, Sweden

**Correction to: Scandinavian Journal of Trauma, Resuscitation and Emergency Medicine (2023) 31:88**

10.1186/s13049-023-01128-3

Author Amir Khorram‑Manesh’s affiliation with the “Center for Disaster Medicine, Gothenburg University, Sweden” was inadvertently omitted from the original publication.

This is re-inserted to the article as affiliation #4 and subsequent co-authors’ affiliations are renumbered to a total of 17.

Co-author Shailly Gupta is affiliated with the “Division of Trauma Surgery & Critical Care, J.P.N. Apex Trauma Center, All India Institute of Medical Sciences, New Delhi, India” and not with the “Joint Centre for Disaster Research, Massey University, Wellington, New Zealand” as initially published.

Correct affiliations are included in this Correction article. The original article has been updated.

